# An instrument for the evaluation of muscle dysmorphia: The Italian validation of the adonis complex questionnaire

**DOI:** 10.1002/brb3.1666

**Published:** 2020-05-29

**Authors:** Giulia Riccobono, Assunta Pompili, Carla Iorio, Giorgio Carducci, Serena Parnanzone, Giulia Pizziconi, Angela Iannitelli, Francesca Pacitti

**Affiliations:** ^1^ Department of Biotechnological and Applied Clinical Sciences University of L'Aquila Coppito (AQ) Italy

**Keywords:** adonis complex questionnaire, bigorexia, body image, muscle dysmorphia, reverse anorexia

## Abstract

**Background and aims:**

Muscle dysmorphia (MDM), or bigorexia, is a subcategory of body dysmorphic disorder (BDD), also known as “Adonis Complex” in nonscientific contexts. One of the most used tools to investigate MDM is the Adonis Complex Questionnaire (ACQ). The ACQ is a 13‐item US questionnaire, designed for male subjects only, related to the dissatisfaction and concerns about physical appearance. The aim of the current study was to evaluate the validity of the Italian version of the ACQ.

**Methods:**

The instrument was administered to a sample of 322 male adults, recruited from the general population. We used the maximum‐likelihood confirmatory factorial analysis (CFA), analyzing the covariance matrices with AMOS 24.0, to evaluate the different factorial models proposed in the literature.

**Results:**

The evaluation of the factorial structure of the Italian version of the ACQ demonstrates the greater stability and internal consistency of the two‐factor model, compared to the original three‐factor model. The factors have no correlation with the demographic characteristics of the sample.

**Conclusions:**

The present study confirms the validity and the reliability of the Italian two‐factor version of the ACQ and highlights the general tendency, among Italian males, to have concerns about their own physical appearance with recurring thoughts and eating behaviors finalized to improve it. Our study represents an advance in the use of adequate and reliable instruments to assess concerns about physical appearance in the Italian male population.

## Graphical abstract

The Italian validation of the ACQ represents an advance in the use of adequate and reliable instruments to assess concerns about physical appearance in the Italian male population.

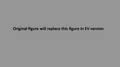

## 1 INTRODUCTION

The body image is a multidimensional construct characterized by the individual's perceptions and assessments of the own physical appearance (Cash, Fleming, Alindogan, Steadman, & Whitehead, 2002). A dysfunctional and/or distorted relationship with body image is related to different types of mental disorders, ranging from the emotional, identity, and relational ones to the psychiatric pathologies, such as anxiety disorders, delusions, suicidal thoughts, substance abuse, deviant behavior, and obsessive–compulsive spectrum symptoms (Phillipou & Castle, 2015; Riccobono et al., 2020; Riccobono, Pompili, Pompili, Iannitelli, & Pacitti, 2019; Ruffolo, Phillips, Phillips, Menard, Fay, & Weisberg, 2006). Mass media and social networks, in recent years, have contributed to the spread of increasingly high aesthetic standards and may have exacerbated some features or even directly stimulated psychological and behavioral contents in predisposed subjects (Dryer, Farr, Farr, Hiramatsu, & Quinton, 2016; Leit, Gray, Gray, & Pope, 2002; Pope, Olivardia, Olivardia, Gruber, & Borowiecki, 1999).

Muscle dysmorphia (MDM), or bigorexia, is a subcategory of body dysmorphic disorder (BDD), also known as “Adonis Complex” in nonscientific contexts; in the past, this syndrome was also known as dysmorphophobia. This syndrome, primarily defined “Reverse Anorexia Nervosa” by Pope, Katz, Katz, and Hudson (1993) and subsequently renamed “Muscle Dysmorphia” (Phillips, O'Sullivan, O'Sullivan, & Pope, 1997), was described as a pathologic preoccupation with muscularity and leanness; subjects affected consider themselves small and frail and perceive their bodies to be significantly leaner and less muscular than they actually are. Other characteristic features are that subjects with MDM typically exercise compulsively, present deviant eating patterns, stare in the mirrors excessively, and many chronically use anabolic–androgenic steroids and other substances in order to get bigger (Baghurst & Lirgg, 2009; Griffiths, Foster, Foster, & Shorter, 2015; Griffiths, Murray, Murray, & Touyz, 2013; Klimek & Hildebrandt, 2018); they also avoid places where their bodies will be seen, such as beaches and swimming pools. These symptoms cause clinically significant distress or impairment in the social and occupational functioning.

In Italy, both for the lack of information about MDM and for reasons of taboos, as elsewhere, this syndrome's topic has only recently been discussed; a recent estimate suggests that Italians affected by muscle dysmorphia are around 60.000 (Longobardi, Prino, Prino, Fabris, & Settanni, 2017). This syndrome is more prevalent in males than in females. Recent researches estimated the prevalence of MDM in the university student population between 5.9% and 6.99% (Bo et al., 2014; Compte, Sepulveda, Sepulveda, & Torrente, 2015).

Subjects with muscle dysmorphia rarely reach clinician's attention, because the MDM it is an ego‐syntonic disorder. Furthermore, MDM it is coherent with the cultural standards and because of that it can be allowed, or even encouraged, by the environment. In fact, these subjects could appear fit, tonic, and successful to others (Leone, Sedory, Sedory, & Gray, 2005), but secretly have concerns and obsessions about their own physical appearance. These factors contribute to make muscle dysmorphia very difficult to study and to treat (Strother, Lemberg, Lemberg, Stanford, & Turberville, 2012).

The tripartite influence model of body dissatisfaction states that social pressure from family, peers, and media leads to the development of body dissatisfaction through the internalization of a societal body ideal and the encouragement of social comparison (Cafri et al., 2005). Tylka (2011) adapted this model to the male population demonstrating that mesomorphic internalization, muscularity, and body fat dissatisfaction play a mediational role in the association between social pressure and disordered eating behaviors. Muscularity and thinness oriented internalization can be considered a potential risk factor for eating disorders in the male population. Longitudinal and cross‐sectional studies, in the adult and adolescent male population, also demonstrated the role of muscularity and eating disorders symptoms in the association between internalization of appearance ideals and body dissatisfaction (Brown, Forney, Forney, Pinner, & Keel, 2017; Cahill & Mussap, 2007; Edwards, Tod, Tod, Molnar, & Markland, 2016). A recent study (Klimek, Murray, Murray, Brown, Gonzales Iv, & Blashill, 2018) pointed out that body ideal internalization in men may be best conceptualized as interactions between muscularity and thinness internalization in association with various disordered eating cognitions and behaviors, such as MDM.

One of the most used tools to assess MDM is the Adonis Complex Questionnaire (ACQ); the ACQ is 13‐item US questionnaire, designed for male subjects only, related to the dissatisfaction and concerns about physical appearance. It was introduced by Pope, Phillips and Olivardia (2000). The questionnaire evaluates the concerns about physical appearance and related behaviors, but does not provide a detailed information about symptoms severity levels. The score ranges from 0 to 39, where 0 is the total absence of concerns about physical image and 39 suggests a serious pathological condition (Latorre‐Román, Garrido‐Ruiz, Garrido‐Ruiz, & Garcia‐Pinillos, 2014).

The aim of the current study was to evaluate the validity of the Italian version of the ACQ.

## 2 METHOD

### 2.1 Participants

The sample consists of 322 adult males (Tables 1 and 2) aged between 18 and 55, recruited from the general population. An online version of the questionnaire has been distributed to a nonbodybuilder population and in closed thematic groups on social networks to subjects who practice bodybuilding and fitness. No exclusions criteria were applied, except all subjects declared that they do not consider themselves professional bodybuilders.

**TABLE 1:** Weight, height, and BMI of the participants

	Min	Max	Mean	*SD*
Weight (kg)	50	98	74.01	9.814
Height (cm)	162	197	176.54	6.104
BMI	17	31	23.76	2.830

**TABLE 2:** Age ranges

	Frequency	Percent	Valid percent	Cumulative percent
<25	64	19.9	19.9	19.9
26–35	82	25.5	25.5	45.3
36–45	95	29.5	29.5	74.8
>46	81	25.2	25.2	100
Total	322	100.0	100.0	

### 2.2 Procedure

The questionnaire has been translated into Italian, with the permission of the author, by two psychiatrists and then translated back to English to verify the reliability of the translation. All the subjects completed the Italian version of the ACQ between June and October 2017. The ACQ consists of 13 items, and the subjects are asked to choose between three possible answers the most representative of their own condition.

The answers indicate in increasing order the presence and severity—from normal to pathological—of the concerns about physical appearance, as well as how this affects life and behavior in personal, social, relational, sexual, nutritional, and sportive life. In particular, values of 0–9 indicate minor concern for body image, 10–19 indicate mild to moderate, 20–29 indicate a serious concern for body image, and, lastly, 30–39 are indicative of severe forms of body image dissatisfaction and MDM (Leone et al., 2005).

### 2.3 Statistical analysis

The factorial analysis of the items, introduced by Latorre‐Román et al. (2014), highlighted three dimensions obtained from three subscale scores, corresponding to 3 internal factors such as: factor 1, psychosocial impact of physical appearance; factor 2, control of physical appearance; and factor 3, concern for physical appearance.

We used the maximum‐likelihood confirmatory factorial analysis (CFA), analyzing the covariance matrices with AMOS 24.0, to evaluate the different factorial models proposed in the literature.

We performed the three‐factor model (3F), primarily introduced by Latorre‐Román et al. (2014), with the 13 items: the first factor consists of items that evaluate the psychosocial impact of physical appearance; the second factor consists of items that investigate the control of physical appearance; and the third factor evaluates the concern for physical appearance.

Moreover, the unidimensional nature of the questionnaire has been evaluated with a one‐factor model for all the 13 items (1F) and with a two‐factor model (2F); the first factor consists of items 2, 3, 8, 9, 10, 11 and the second factor includes items 1, 4, 5, 6, 7, 12, 13.

A confirmatory factor analyses (CFA) were conducted to assess the underlying factor structure of the ACQ. The model fit was assessed using root mean square error of approximation (RMSEA), and values <0.06 were considered a good fit. The validation analysis of the models was evaluated through the two indices method.

The chi‐square and the comparative fit index (CFI) were used to compare the accuracy of the models.

We conducted an exploratory factor analysis, in order to explore the factorial structure of the ACQ in the sample, using the principal component analysis (PCA) method and the varimax rotation on the 13 items. Factor correlations were interpreted using the factorial correlation matrix.

The suitability of the data for the factor analysis was assessed using the spherical test of and the value of Kaiser–Meyer–Olkin. We used the Kaiser–Guttman criterion, supported, where necessary, by the Cattell Scree Test to choose the number of factors. The reliability of the questionnaire and the homogeneity of the factors were evaluated calculating Cronbach's *α* coefficient for each factor and the mean of the interitem correlations.

### 2.4 Ethics

This research was conducted in accordance with the Declaration of Helsinki and was approved by the ethical Committee of the University of L'Aquila. All the procedures were carried out with the adequate understanding of the subjects, who read and signed an informed consent form before participating in this research project. All the authors declare that no financial support was received for this study.

## 3 RESULTS

The 322 subjects have a mean age of 38.1 years (*SD* = 11.28). As regards of the educational level, the majority of the participants, 125 subjects (38.8%), has high school graduation, 71 (22%) have a 6‐year university degree, 58 (18%) have a 3‐year university degree, 57 (17.7%) have another postgraduate degree, and 11 (3.4%) have a secondary school diploma. The 85.4% of the participants presented minor concerns about physical appearance, the 13.7% mild or moderate concerns, and the 0.9% serious (Table 3). The subjects aged between 36 and 45 years presented higher percent of mild or moderate concerns (18.9%) (Table 4).

**TABLE 3:** Severity levels

	Frequency	Percent	Valid percent	Cumulative percent
Minor concern	275	85.4	85.4	85.4
Mild or moderate	44	13.7	13.7	99.1
Serious	3	0.9	0.9	100.0
Total	322	100.0	100.0	

**TABLE 4:** Age ranges and severity levels

Age range	Severity	Frequency	Percent	Valid percent	Cumulative percent
<25	Minor concern	56	87.5	87.5	87.5
	Mild or moderate	8	12.5	12.5	100
	Total	64	100	100	
26–35	Minor concern	72	87.8	87.8	87.8
	Mild or moderate	9	11.0	11.0	98.8
	Serious	1	1.2	1.2	100
	Total	82	100	100	
36–45	Minor concerns	76	80	80	80
	Mild or moderate	18	18	18.9	98.9
	Serious	1	1.1	1.1	100
	Total	95	100	100	
>46	Minor concerns	71	87.7	87.7	87.7
	Mild or moderate	9	11.1	11.1	98.8
	Serious	1	1.2	1.2	100
	Total	81	100	100	

The CFA has shown that the one‐factor model (1F) is not suitable, while both the two (2F)‐ and three‐factor (3F) models can be accepted (Table 5). The suitability of the data for the factor analysis was assessed using the spherical test of Bartlett (*x* (78) = 1,172, *p* < .001), and the value of Kaiser–Meyer–Olkin measure of sampling adequacy was 0.841.

**TABLE 5:** Correlations

Factors	*χ* ^2^	*df*	SRMR	RMSEA	HI90	LO90	CFI
1	447	65	0.109	0.135	0.147	0.124	0.65
2	114	55	0.064	0.058	0.073	0.043	0.95
3	143	57	0.072	0.069	0.083	0.055	0.92

However, the two‐factor model, from the analysis of the indices, obtained good fit values in SRMR, CFI, and chi‐square. The choice of the two‐factor model was confirmed by the correlation analysis. While in both models the *psychosocial effect of physical appearance* and the *control of physical appearance* factors correlate moderately (*r* = .41), as well as *concern for physical appearance* and the *psychosocial effect of physical appearance* factors in the 3F model (*r* = .44), t*he control of physical appearance* and the *concern for physical appearance* factors in the 3F model show a high correlation (*r* = .85), suggesting they belong to a single factor. To confirm the two‐factor solution, was conducted an exploratory factorial analysis (EFA) using PCA with varimax rotation, that produced a two‐factor solution (47.78% of the variance), corresponding to the 2F model tested with the CFA.

The first factor consists of seven items, related to the *psychosocial effect of physical appearance*, and explains 24.77% of the variance. The second factor consists of 6 items, related to *control of physical appearance*, and explains 23.01% of the variance (Table 6). The reliability of the ACQ and the homogeneity of factors were satisfactory for both the factors (Table 7). The correlation analysis between the ACQ factors and the demographic variables did not show significant relations.

**TABLE 6:** Factorial models

Items	1F model	2F model		3F model		
	F1	F1	F2	F1	F2	F3
1: “How much time do you spend each day worrying about some aspect of appearance (not just thinking about it, but actually worrying about it)?”	0.551	0.551		0.551		0.459
2: “How often are you distressed by your appearance concerns (that is feeling upset, anxious or depressed)?”	0.602	0.602		0.602		
3: “How often do you avoid having all or part of your body seen by others? For example, how often do you avoid locker rooms, swimming pools or situations where you have to take your clothes off? Alternatively, how often do you wear certain clothes to alter or disguise your body appearance‐ such as a hat to hide your hair or baggy clothes to hide your body?”	0.483	0.483	0.526	0.483	0.526	
4: “How much total time do you spend each day involved in grooming activities to improve your experience?”	0.519	0.519	−0.359	0.519	0.359	0.538
5: “How much total time do you spend each day on physical activities to improve your body appearance, such as lifting weights, doing sit‐ups, or running on a treadmill?”	0.566	0.566	−0.500	0.566	−0.500	
6: “How often do you engage in dieting, eating special foods (for example high protein or low‐fat foods), or taking nutritional supplements specially to improve your appearance?”	0.530	0.530	−0.392	0.530	−0.392	−0.343
7: “How much of your income do you spend on items designed to improve your appearance (for example diet food, nutritional supplements, hair products, cosmetics and cosmetic procedures, workout equipment or gym memberships)?”	0.591	0.591	−0.367	0.591	−0.367	
8: “How much have your appearance related‐activities undermine your social relationships? For example, have your workout activities, dietary practices, or other appearance‐related behaviors compromised your relationships with other people?”	0.629	0.629		0.629		
9: “How often has your sex life been compromised by your appearance concerns?”	0.514	0.514	0.564	0.514	0.564	
10: “How often have appearance‐related concerns or activities compromised your job or career (or academic performance if you are a student)? For example, have you been late, miss work or school, worked below your potential or lost opportunities for advancement because of your appearance‐related needs or self‐consciousness?”	0.623	0.623	0.371	0.623	0.371	
11: “How often have you avoided being seen by other people because of your appearance concerns (for example, not going to school, work, social events, or out in public)?”	0.586	0.586	0.543	0.586	0.543	
12: “Have you ever taken any type of drug‐ legal or illegal‐ to gain muscle, lose weight, or otherwise improve your appearance?”	0.543	0.543	−0.367	0.543	−0.367	−0.449
13: “How often have you used more extreme measures (other than drug use) to change your appearance, such as excessive exercising; working out even when injured; fasting or other unhealthy dietary activities; vomiting, use or laxatives or other ‘purging’ methods; or unconventional techniques for muscle development, hair growth, penile enlargement, etc..?”	0.630	0.630		0.630		−0.312

**TABLE 7:** Internal consistency reliability (Cronbach's *α*) of the ACQ

	No. of item	Mean	*SD*	Cronbach's *α*
ACQ	13	4.84	4.5	.812
Psychosocial effect of physical appearance	6	2.22	2.47	.781
Control of physical appearance	7	2.61	3	.772

## 4 DISCUSSION

The Adonis Complex concept (Pope et al., 2000) refers to general aspects related to male body dissatisfaction, including other body dissatisfactions, such as alopecia or penis size (Pope, Gruber, Choi, Olivardia, & Phillips, 1997; Pope et al., 2000). The ACQ, introduced by Pope, Phillips and Olivardia, is the most used instrument to assess the concerns about physical appearance and related behaviors in the male population.

The structure originally proposed by Pope and the Latorre‐Román Spanish validation found a three‐factor model that was not confirmed in our sample. A recent study (Sepulveda, Rica, Rica, Moreno, Roman, & Compte, 2019) administered the ACQ in a population of male university students highlighting a not acceptable fit of the three‐factor model and proposed a one‐factor structure, showing an adequate level of internal consistency, with Cronbach's *α* of .90, higher than the study of Latorre‐Román, Garrido‐Ruiz, and García‐Pinillos (2015) . We wonder whether the appropriateness of the three‐factor model, reported in the Spanish validation of Latorre‐Román, is due to the characteristics of the sample—composed exclusively of bodybuilders—while in the present study and in Sepulveda's, the questionnaire was administered to a nonbodybuilder population.

The evaluation of the factorial structure of the Italian version of the ACQ, carried out through an exploratory and confirmatory factor analysis, demonstrates the greater stability of the two‐factor model, compared to the original three‐factor model proposed by Latorre‐Román et al. (2014). In this case, the first factor consists of items that evaluate the *psychosocial effect of physical appearance*, and the second both the c*oncern for the physical appearance* and the *control of physical appearance*, highly interrelated. Both the factors show a good internal consistency.

The *concern* and the *control of physical appearance* factors are closely related to each other, and this finding would support the literature that considers body dysmorphic disorder (BDD) as belonging to the obsessive–compulsive spectrum.

The significantly higher values in the answers, taken individually, are those concerning the food behaviors (50.3% of subjects sometimes or frequently use a diet and/or take food supplement to improve their physical appearance), the stress for the physical appearance (48.7% of subjects sometimes or frequently are worried and/or anxious/stressed), and the avoidance of body exposure (39.5% of subjects sometimes or frequently avoid places where they are expected to take off clothes like changing rooms or pools, or hide their body with large clothes or hats). These findings, together with the noncorrelation with the demographic characteristics, show that there is a general tendency to have concerns about the physical appearance with recurring thoughts and eating behaviors finalized to improve it, regardless of the age and the educational level, as other studies on this topic demonstrated (Phillips, Gunderson, Gunderson, Mallya, McElroy, & Carter, 1998; Phillips, McElroy, Hudson, & Pope, 1995). In this way, in a mixed population (not exclusively sportsmen and/or bodybuilders) dietary behavior is considered the most used method to control the physical appearance, searching for physical fitness probably through food restrictions (Murray et al., 2012). This can be the reason why, in the DSM‐5, muscle dysmorphia and body dysmorphic disorder (BDD) are considered a bridge between the obsessive–compulsive spectrum disorders and the eating behavior disorders; this finding can have some major clinical implications.

The findings of the current research confirm that the features of the two disorders are present also among the general Italian male population. It would be interesting, in further studies, to carry out a comparative research administering the questionnaire to a population of bodybuilders and sportsmen in order to highlight any significant differences.

### 4.1 Study limitations

Despite its contributions, this study has some limitations that should be considered. Firstly, the CFA was performed on the same EFA's sample. Generally, EFA and CFA should be performed on separate samples or on two distinct subsamples from the same sample. However, given the relatively small sample size, splitting the sample in two halves would have result in a small sample size that could have limited statistical power. Secondly, a strong limitation is that other questionnaires, such as Exercise Dependence Scale‐Revised (EDS‐R) and Eating Attitudes Test (EAT‐26), could be administered to evaluate concurrent validity.

Another limitation is that the ACQ was administered only to a male population, and it would be interesting, in future researches, to analyze the psychometric properties of the Italian version of the ACQ also in a female sample.

## 5 CONCLUSION

The current study confirms the validity and the reliability of the Italian two‐factor version of the ACQ and highlights the general tendency, among Italian males, to have concerns about their own physical appearance with recurring thoughts and eating behaviors finalized to improve it.

Our study represents an advance in the use of adequate and reliable instruments to assess concerns about physical appearance in the Italian male population.

## CONFLICT OF INTEREST

The authors declare no potential conflict of interest.

## AUTHOR CONTRIBUTIONS

GR has been involved in the study concept and design, in the review of the literature, in the data analysis and interpretation, and in the drafting and revision of the manuscript. AP has been involved in the study supervision, in the supervision of the methodology, and in the review and editing of the manuscript. CI has been involved in the data curation, and in the review and editing of the manuscript. GC has been involved in the in the data collection and in the review of the literature. GP has been involved in drafting and reviewing the manuscript. SP has been involved in drafting and reviewing the manuscript. AI has been involved in the study supervision, in the supervision of the methodology, and in the review and editing. FP has been involved in the conception and design of the study, in the data analysis and interpretation, and in the drafting and revision of the manuscript. Moreover, she supervised all of the steps of the study (review of literature, data analysis, interpretation data, drafting/revision of the manuscript). All of the authors agreed on the order in which their names have been listed and gave the final approval of the version to be published.

## Data Availability

The data that support the findings of this study are available from the corresponding author upon reasonable request.
